# Simulating price subsidies on healthy foods in Mexico

**DOI:** 10.1017/S1368980024002702

**Published:** 2025-03-24

**Authors:** Jesús E Morales-Ríos, Mishel Unar-Munguía, Carolina Batis, Josué A Quiroz-Reyes, Néstor A Sánchez-Ortiz, M Arantxa Colchero

**Affiliations:** 1 Center for Research on Evaluation and Surveys, Instituto Nacional de Salud Pública, 62100 Cuernavaca, Morelos, Mexico; 2 Center for Nutrition and Health Research, Instituto Nacional de Salud Pública, 62100 Cuernavaca, Morelos, Mexico

**Keywords:** Suboptimal consumption, Purchases, Prices, Elasticities, Subsidy

## Abstract

**Objective:**

To simulate the impact of a price subsidy (price reduction) on purchases of healthy foods with suboptimal consumption.

**Design:**

We used data from the 2018 Mexican National Household Income and Expenditure Survey, a cross-sectional study. We estimated own- and cross-price elasticities of the demand for food groups using a Linear Approximation of an Almost Ideal Demand System. Using the estimated elasticities, we derived changes in purchases associated with a 10, 20 and 30 % price reduction in healthy food groups with suboptimal consumption. We also estimated price reductions for these food groups that would meet the recommendations of the Healthy Reference Diet (EAT-HRD) proposed by the EAT-Lancet commission.

**Setting:**

Mexico (country).

**Participants:**

A nationally representative sample of mexican households.

**Results:**

Price reductions were associated with increases in the quantity purchased, ranging from 9·4 to 28·3 % for vegetables, 7·9 to 23·8 % for fruits, 0·8 to 2·5 % for legumes and 6·0 to 18·0 % for fish. Higher reductions in prices would be needed to achieve the EAT-Lancet Commission’s recommendations for food groups with suboptimal consumption in Mexico: a 39·7 % reduction in prices for fruits, 20·0 % for vegetables and 118·7 % for legumes.

**Conclusions:**

Our study shows that reductions in prices can lead to increases in purchases of healthier food options. More research is needed to assess the most cost-effective strategy to deliver subsidies using either conditional cash transfers, vouchers or food baskets provided to families or direct subsidies to producers.

Overweight and obesity represent a public health problem in most nations. In 2016, the WHO estimated that 1900 million adults were overweight and 650 million were obese^(1)^. In Mexico, the prevalence of overweight and obesity in 2020 was 76 and 72·1 % for women and men, respectively^(2)^. Conditions associated with obesity, including CVD such as hypertension, IHD, acute myocardial infarction and metabolic diseases such as diabetes, represent a significant burden for the Mexican health system^(3–6)^. Obesity has a multifactorial origin that involves family history, physical activity, intestinal microbiota, genetic alterations, epigenetic modifications and most importantly diet^5^.

Diet is a potentially modifiable risk factor for overweight and obesity, as well as their complications^(7)^. Although there are several recommendations to improve diet, the Healthy Reference Diet (EAT-HRD) proposed by the EAT-Lancet commission considers both the impact on health and the environment in the production of certain foods^(8)^. In the USA, a more sustainable dietary pattern was associated with lower odds of obesity in adults^(9)^. The EAT-HRD was proposed as a global effort to integrate healthy nutrition and sustainable food production. The EAT-HRD recommends a plant-based diet with a variety of foods including fruits, vegetables, whole grains, legumes and nuts and with limited consumption of red and processed meats and sugar. The EAT-HRD is a flexible guide that can be adapted to the needs, tastes and culture of a country and reduces the burden of unhealthy diets for worldwide malnutrition, obesity and climate change^(8,10)^.

The EAT-HRD recommendations for food consumption are based on global environmental and health goals. Based on the EAT-HRD recommendations, the Mexican population exceeds the consumption of refined grains, corn, dairy products, added sugars, animal proteins and eggs. In contrast, there is a suboptimal consumption (below EAT-HRD recommendations) for fish (0·5 times less compared with the recommendation), 0·7 less for high-fibre grains (excluding corn), 0·7 less for fruits, 0·8 lower for vegetables, 0·3 for legumes and 0·03 times less for nuts. The EAT-HRD suggests a daily intake of 300 g for vegetables (with a range between 200 and 600 g), 200 g of fruits (ranging from 100 to 300 g), 100 g of legumes (from 0 to 225 g) and 28 g of fish and seafood (ranging from 0 to 100 g)^(11)^.

Diet is determined by a complex relationship between environmental and socio-economic factors. These factors include the food supply, health promotion, food marketing, prices, place of residence, education and household income^(12–14)^. In particular, prices largely determine food choices, and income represents the budgetary constraint to purchase food.

Subsidies can be used to incentivise the consumption of healthy foods or foods with low consumption by reducing their price to increase their purchases. Subsidies can be implemented indirectly through the application of agricultural subsidies, livestock, poultry or fish production, but more often, subsidies have been applied directly through the distribution of discount coupons, monetary transfers or food baskets to families^(15)^.

There is evidence in some countries that implementing healthy food subsidies can increase the intake of healthy foods and improve dietary patterns as reported in simulations and experimental designs^(16,17)^. Many studies report that subsidies such as conditional cash transfers and discount vouchers for fruits and vegetables increase the consumption of these foods, which may lead to less cardiovascular risk and death prevention^(18–27)^. In addition, subsidised healthy food baskets increase the consumption of fruits and vegetables among children and improve diet quality and malnutrition biomarkers^(28)^. Experimental designs have shown that people choose and increase healthy food purchases when faced with a price reduction^(19,21,22)^. Simulation studies replicate these findings and propose a combination of subsidised healthy foods with taxes on unhealthy foods. Many of these studies come from high-income countries that may not be generalised to low- or middle-income countries given differences in consumption patterns, supply chain and food supply^(29)^.

In Mexico, several public policies have been implemented to discourage the consumption of unhealthy foods such as front-of-pack labelling and marketing regulations to children and to food offered in public schools^(30–32)^. In addition, specific taxes on sugar-sweetened beverages and non-essential energy-dense food have been implemented and have been shown to be effective in reducing consumption^(33)^.

However, strategies to incentivise the consumption of healthy foods such as subsidies have not been explored. The aim of this study was to simulate the impact on purchases of a price subsidy (price reduction of 10, 20 and 30 %) on healthy foods with suboptimal consumption (fruits, vegetables, legumes and fish and seafood) in Mexico using a nationally representative survey. We also estimated the percentage of price reduction that would be required to meet the EAT-HRD recommendations for these foods.

## Methods

### Data

We used data from the 2018 Mexican National Household Income and Expenditure Survey (MNHIES), which has a probabilistic two-stage clustered design and is representative at the national level^(34)^. The MNHIES includes data on occupational and sociodemographic characteristics at the individual level and income and expenditures at the household level. Expenditures and the quantity purchased of household food are recorded for seven consecutive days. The survey also reports food away from home expenditures, but the specific food items and quantity purchased are not reported.

### Empirical model

We estimated a demand system for thirteen food and beverage groups using a Linear Approximation of an Almost Ideal Demand System (LA/AIDS) developed by Deaton and Muellbauer^(35)^. The model estimates price elasticities for each food group included using simultaneous equations. Demand systems replicate how consumers purchase food and beverages by simultaneously considering relative prices (prices of all goods) and resource constraints such as income, which is consistent with economic theory. Thirteen demand equations were estimated for each group, adjusting for household demographic and economic covariates. The model specification is as follows:
(1)

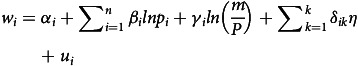

where 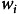 is the share or proportion of food expenditure of the 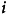 -th food group, 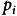 is the mean nominal price of the 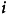 -th food group, 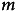 is the total household food expenditures, 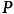 corresponds to the Stone’s price index, 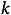 denotes the number of covariates at household level and 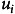 is the random error term. Stones’ price index is defined as 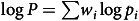 that captures the expenditures and relative prices among the food groups and households. The model needs to comply with the following: additivity 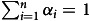 , 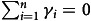 , 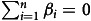 , homogeneity 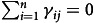 and symmetry 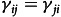 restrictions.

Marshallian or uncompensated price elasticities were estimated at the means of each covariable. Price elasticities measure how much the consumption of a good changes if the price increases. A good is inelastic if the own-price elasticity is less than 1 (in absolute terms since own-price elasticities are expected to be negative), which means that the percentage reduction in consumption is lower compared with the proportional price increase, and it is elastic if the elasticity is greater than 1. Basic foods are generally price inelastic, while ultra-processed foods and some non-basic healthy foods tend to be more elastic. Price elasticities are defined as follows:
(2)

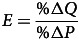




where 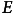 corresponds to the own-price elasticity of demand, 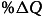 is the percentage change in the quantity purchased and 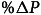 is the percentage change in the prices of each food group.

Using the estimated price elasticities, we derived changes in the quantity purchased using own- and cross-price elasticities for each food group with suboptimal consumption to account for the whole effect of a simultaneous price reduction in these food groups. We modelled three scenarios of price reductions: 10, 20 and 30 %. From equation 2, the quantity purchased after the price reduction was estimated as:

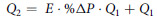

where 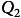 is the average quantity purchased after the price reduction, 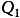 is the initial average quantity purchased and 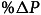 is the 10, 20 and 30 % percentage change.

As the EAT-HRD recommendations are different for each food, a differential price reduction may be needed. In addition to the 10, 20 and 30 % price reductions simulations, we estimated the percent price reduction that would be required to meet the EAT-HRD recommendations for a healthy diet that is 200 g per d for fruits, 300 g per d for vegetables, 100 g per d for legumes and 28 g per d for fish and seafood. We calculated the mean purchase *per capita* for each food with suboptimal consumption by dividing mean purchases per household by mean household size, then this weekly purchase *per capita* is divided by seven to obtain the mean purchase for each individual per d.

### Food and beverage groups

The MNHIES reports 242 food or beverage items, which are reported in single groups for the most consumed foods (such as tomatoes, zucchini, bananas) or grouped for less consumed items (cherries, blackberries and raspberries reported as a single item). For this analysis, the 242 food items were grouped into thirteen groups according to their nutritional nature: fruits, vegetables, legumes, cereals and seeds, eggs, dairy products, unprocessed meat, fish and seafood, water, processed meats, prepared foods, taxed food and beverages (sugary drinks and non-basic energy-dense foods) and other foods that did not fall into the previous categories (food and beverages included in each group are described in online supplementary material, Supplemental Table 1).

### Variables

The dependent variable is the proportion or share of the total weekly food expenditure (as reported in the survey) spent on a particular food group. This proportion is calculated by dividing the weekly expenditure on that specific food group by the total weekly expenditure on all thirteen food groups combined. The independent variable is the price of food expressed in Mexican pesos per litre of beverage or kilogram of food. Prices were derived from the sum of the amount spent divided by the sum of the quantity of food purchased in a food group by municipality. We aggregated prices at the municipality level to reduce potential recall bias in the quantity purchased or in expenditures. In the absence of a municipal average price, the national average was assigned. Prices were deflated to 2023 Mexican pesos.

Given that the shares of expenditures for each food groups could be associated with different factors, the model was adjusted for household sociodemographic and economic characteristics. Area of residence was defined as rural (localities with up to 2500 inhabitants) or urban (localities with more than 2500 inhabitants). Education of the head of the household corresponds to the last level of education formally completed: no schooling, primary, secondary, high school and university or higher. From quarterly household income, we created income quintiles: very low, low, medium, high and very high. Finally, we included household size and household composition as binary variables for the presence of children aged 0–1, 2–5, 6–13, women aged 14–18, men aged 14–18, women over 18 and men over 18 years. Households without demographic information or no food or beverage expenditures were excluded. We used Stata version 18.0 (StataCorp LLC) software and the survey module to incorporate the complex survey design.

## Results

The analytical sample included 74 647 households, of which 1·1 % were excluded for lack of demographic information or no expenditures in any of the food groups (see online supplementary material, Supplemental Figure 1).

Table 1 shows household sociodemographic and economic characteristics. Almost 77 % of households are urban. On average, households are made up of 3·6 members with a greater presence of men and women over 18 years of age. The largest proportion of the head of households have a high school education and a smaller proportion have a bachelor’s or graduate degree.

**Table 1.** Sociodemographic and economic characteristics of households

Variable	Average or proportion
	MNHIES 2018 *n* 73 811
Household type	
Urban	77·1 %
Household size	3·6
Household composition	
Children 0–1 years	9·6 %
Children 2–5 years	20·8 %
Children 6–13 years old	36·2 %
Males 14–18 years old	15·4 %
Females 14–18 years old	15·3 %
Males > 18 years old	84·8 %
Women >18 years old	91·7 %
Schooling of the head of household	
No schooling	21·4 %
Primary	19·8 %
Secondary	29·3 %
High school	15·7 %
University or higher	13·9 %
Quarterly income per quintile (Mexican pesos of 2023)	
Very low	12 030·7
Low	22 848·8
Medium	33 813·6
High	50 204·2
Very high	116 791·5

Own estimations using the 2018 MNHIES.

Over 80 % of households purchased vegetables and cereals, while water, fish and seafood are the least purchased groups (Table 2). Households allocate most of their spending on purchases of unprocessed meats, dairy products, vegetables and taxed food and beverages. Water and legumes are the groups with the lowest share of household food expenditures. The groups with the highest price per kilogram are processed and unprocessed meats. In contrast, water and cereals are the groups with the lowest price per kilogram or litre.

**Table 2.** Proportion of households with expenditures greater than zero, distribution of household expenditure and average prices by food group

Percentage of households with expenditures in each food group	Average or proportion
Fruits	52·8 %
Vegetables	83·7 %
Legumes	45·5 %
Cereals and seeds	94·1 %
Egg	64·1 %
Dairy products	79·2 %
Unprocessed meats	72·5 %
Fish and seafood	18·0 %
Water	34·1 %
Processed meats	55·2 %
Prepared foods	43·3 %
Taxed food and beverages	85·7 %
Other foods	58·2 %
Distribution of household food expenditure	
Fruits	4·3 %
Vegetables	11·0 %
Legumes	2·6 %
Cereals and seeds	14·6 %
Egg	4·4 %
Dairy products	10·2 %
Unprocessed meats	16·5 %
Fish and seafood	1·8 %
Water	2·0 %
Processed meats	4·9 %
Prepared foods	9·5 %
Taxed food and beverages	13·7 %
Other foods	4·6 %
Average prices (aggregated at the municipal level in Mexican pesos per kg or L)*	
Fruits	12·8
Vegetables	15·6
Legumes	17·4
Cereals and seeds	11·9
Egg	20·5
Dairy products	19·2
Unprocessed meats	53·9
Fish and seafood	63·9
Water	1·1
Processed meats	56·5
Prepared foods	31·8
Taxed food and beverages	14·7
Other foods	25·2

Own estimations using the 2018 MNHIES. Descriptive statistics used the complex survey design. *2023 Mexican pesos per kg or L of food as purchased (raw with inedible portion).

Coefficients from the demand system are shown in online supplementary material, Supplemental Tables 2 and 3. Table 3 shows the own-price elasticities of demand for each of the thirteen food groups (full price elasticities matrices are shown in online supplementary material, Supplemental Table 4). The elasticity for vegetables is −1·09, showing an elastic demand: a 10 % increase in price is associated with a proportionally higher reduction in purchases of 10·9 %. Fruits and legumes are more inelastic with a price elasticity lower than 1 (in absolute terms): −0·73 and −0·38, respectively. Fish and seafood are more elastic than the other food groups with suboptimal consumption: a 10 % increase in price is associated with a reduction in purchases of 14·5 %.

**Table 3.** Own-price elasticities of food groups

Food group	Own-price elasticity	95 % CI
Fruits	−0·74	–0·79, –0·70
Vegetables	−1·09	–1·13, –1·05
Legumes	−0·37	–0·42, –0·31
Cereals and seeds	−0·39	–0·42, –0·36
Egg	−0·69	–0·75, –0·64
Dairy products	−1·34	–1·37, –1·31
Unprocessed meats	−0·87	–0·90, –0·84
Fish and seafood	−1·45	–1·50, –1·40
Water	−1·92	–2·01, –1·84
Processed meats	−0·72	–0·77, –0·68
Prepared foods	−1·03	–1·05, –1·01
Taxed food and beverages	−1·29	–1·33, –1·25
Other foods	−0·57	–0·63, –0·52

Own estimations using the 2018 MNHIES.

**Figure 1.**
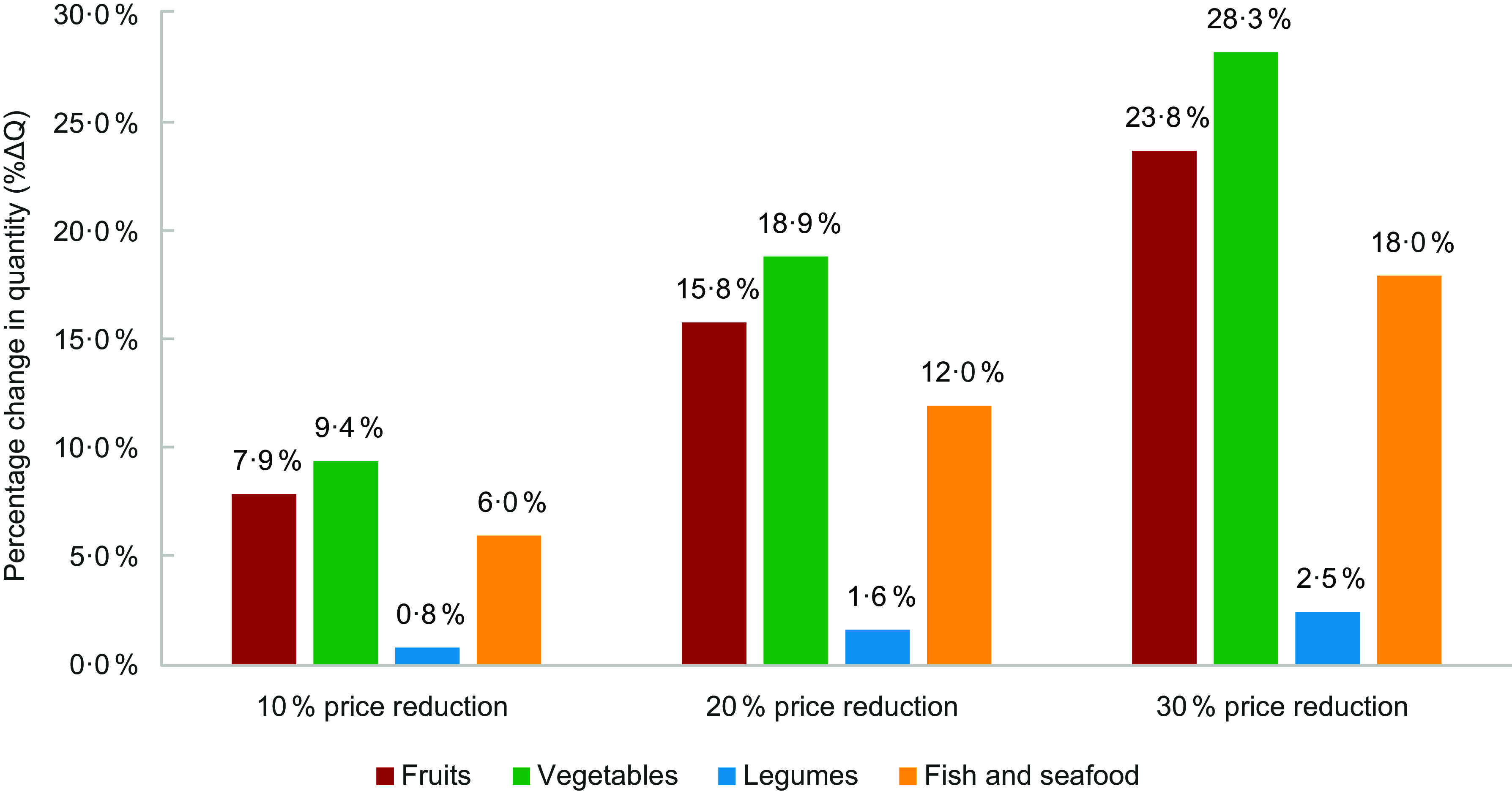
Simulated changes in quantity purchased of fruits, vegetables, legumes and fish and seafood for three scenarios of price reduction (10, 20, 30 %).

The simulation of a price subsidy to healthy food groups with suboptimal consumption is shown in Fig. 1. A 10 % price reduction increases the quantity purchased per household for vegetables by 9·4 %, for fruits by 7·9 %, for legumes by 0·8 % and fish and seafood by 6·0 %. These increases correspond to an increase in 0·68 kg/week for vegetables, 0·28 kg/week for fruits, 0·05 kg/week for legumes and 0·17 kg/week for fish and seafood. A price subsidy of 20 and 30 % would increase food purchases by the same proportion, assuming linearity. For example, if a 10 % reduction in price is associated with a 7·4 % increase in purchases of fruits (167 g) (based on the own-price elasticity for fruits of −0·74), with a 20 % price reduction, purchases would increase 7·4 % more to 178 g.

Actual mean *per capita* daily purchases of fruit are 154 g, 246 g of vegetables, 53 g of legumes and 47 g of fish. With a simultaneous 10, 20 and 30 % price reduction, purchases increased by 167 g, 178 g and 191 g for fruits; 269 g, 292 g and 315 g for vegetables; 53·9 g, 54·3 g, 54·7 g for legumes; and 49·7 g, 52·5 g and 55·3 g for fish. However, a price reduction of 39·7 % for fruits, 20·0 % for vegetables and 118·7 % for legumes would be needed to achieve the EAT-HRD recommendations for a healthy diet.

## Discussion

Own-price elasticities of thirteen food groups were estimated using an adjusted LA/AIDS model accounting for household characteristics with the MNHIES 2018. Price reductions (10, 20 and 30 %) were simulated for healthy food groups with suboptimal consumption (fruits, vegetables, legumes and fish), and changes in purchases were estimated using price elasticities. Price reductions resulted in increases in purchases for all healthy food groups, but differential price decreases would be needed to meet the EAT-HRD recommendations^(8)^.

Price elasticities for the suboptimal consumed food groups were of expected direction based on published literature and are low in comparison with other food groups such as dairy products, fish and seafood, prepared foods and taxed products (see online supplementary material, Supplemental Table 5). Several studies have estimated price elasticities of these groups in different contexts and populations and match to a great extent with our results.

Our findings are consistent with studies using demand system models such as the one used in this study, which simulated price reductions using national survey data. Valizadeh and colleagues estimated that a price reduction in fruits and vegetables increases the consumption of these foods^(36)^. Likewise, a study in the USA that used the 2009–2010 National Health and Nutrition Examination Survey estimated that just a 10 % reduction in prices of healthy foods would increase the consumption of healthy meals by 2·04 % for men and 0·74 % among women and would reduce unhealthy meals consumption by –2·74 % for men and by –1·04 % for women^(37)^. Also, Lin and colleagues, who estimated price elasticities for twelve foods that are consumed often in breakfast in the USA, reported that these foods were inelastic and a price reduction of 10 % in high-nutrition ready-to-eat breakfast cereals would increase total calories at breakfast without a substantial increase in added sugars^(24)^.

Nordström *et al.* reported that the elimination of the value-added tax on healthy foods and the application of a specific tax on unhealthy foods increased the consumption of healthy foods, especially high-fibre grains. However, they found an increase in the consumption of unhealthy nutrients such as fats, salt and added sugars among low-income households because of the available substitute foods, which are high in those nutrients^(25,26)^. Despite these results, they found that the increase in fruit and vegetables can lead to the prevention of more than 6400 CVD and cancer-related deaths per year^(27)^.

Evaluation of subsidy programmes, including conditional cash transfers or discount coupons, has reported increases in the consumption of fruits and vegetables^(18–27,38)^. For instance, aboriginal families in North Wales who received these baskets increased the consumption of fruits and vegetables and improved their Hb levels, particularly among children^(39)^. A controlled study conducted in a US cafeteria, where clients had a cash or voucher subsidy, along with food and anti-obesity advertising, reported a reduction in the caloric intake from fats and carbohydrates^(19)^. An experiment in New Zealand, where subjects were exposed to a virtual supermarket for 5 weeks with random price changes as subsidies on fruits and vegetables and taxes on sugar-sweetened beverages or saturated fat and salt, showed that the subsidy intervention group experienced an increase in absolute purchases of fruits and vegetables^(21)^. In Saudi Arabia, a small sample of students from a university exposed to subsidised healthy foods and to taxes on unhealthy items showed that both interventions led to an increase in healthier food choices^(22)^.

Our study has some limitations. The first limitation is the potential recall biases on household expenditures and the quantity purchased. We addressed this drawback by aggregating prices at the municipal level. Another limitation is that we could not include foods and beverages purchased for consumption away from home, which accounts for at least 20 % of total food expenditures^(40)^.

The fiscal feasibility, sustainability and the delivery mechanisms of a subsidy are key aspects of its implementation. As mentioned, subsidies can be applied as cash transfers, discount vouchers or in-kind subsidies such as food baskets. Each of these mechanisms has a different impact on household purchases. Monetary transfers provide the highest welfare as individuals would have an increase in their budget constraint that would allow them to purchase any goods they need; this moves the economy to a new Pareto optimum. In addition, cash transfers have less social stigma associated with government support compared with vouchers or baskets^(41)^.

Direct subsidies to families (through cash transfers, vouchers or food baskets) may be more efficient compared with reductions in prices through subsidies to producers, as they can be targeted to the populations that would benefit more and make healthy food options more available and affordable^(42,43)^. Compared with cash transfers and vouchers, food baskets have a higher distribution cost^(41)^.

Contrary to subsidies, where it is unclear in which subgroups of the population the subsidy has an effect, discount vouchers or food baskets allow targeting the subsidy to households that really require the benefit. In addition, these types of benefits distort the market less, since they supply food, whose consumption is to be increased. Cash transfers, vouchers and in-kind subsidies had a cost per transaction, but cash transfers appear to be less costly and easier to administer than the others^(41,44)^.

Agricultural subsidies increase consumption of healthy food and may lead to positive impacts on health^(20,39,45–49)^. However, the implementation of agricultural subsidies is more complex in economies with trade agreements such as in Mexico. Estimating the costs of implementing subsidies is crucial. Fiscal revenues from existing taxes could be used to provide subsidies as a complementary public health strategy to incentivise the consumption of healthier food options.

### Conclusions

Our study shows that price reductions (through subsidies) are associated with increases in purchases of fruits, vegetables, legumes and fish, which have suboptimal consumption in Mexico. The magnitude of this increase depends on price elasticity: fish and vegetables are more price sensitive and more price elastic compared with fruits and legumes. Moreover, a differential price decrease on the suboptimal consumed foods would meet the EAT-HRD recommendations. More research is needed to assess the most cost-effective strategy to deliver subsidies using either conditional cash transfers, vouchers or food baskets delivered to families or direct subsidies to producers.

## Supporting information

Morales-Ríos et al. supplementary material Morales-Ríos et al. supplementary material

## Data Availability

The data are publicly available at (https://www.inegi.org.mx/programas/enigh/nc/2018/). The analytic code will be made available upon request pending application and approval.

## Supplementary material

For supplementary material accompanying this paper, visit https://doi.org/10.1017/S1368980024002702.
