# Supplementary material for: Simulating price subsidies on healthy foods in Mexico
Source: Public Health Nutr. 2025 Mar 24;28(1):e74. doi: 10.1017/S1368980024002702 (PMC12086732; doi:10.1017/S1368980024002702)
Supplement: Morales-Ríos et al. supplementary material [file S1368980024002702sup001.docx]

**Supplementary Material**

**Supplementary Figure 1.** Analytical sample

**
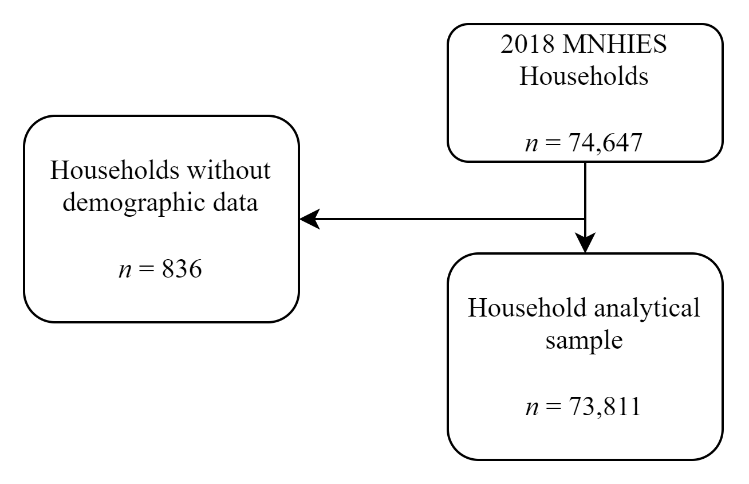
**

**Supplementary Table 1.** Food groups included in the estimation of price elasticities.

| **Group** | **Foods** |
| --- | --- |
| **Fruits** | Anona, custard apple, soursop, cherry, raspberry, strawberry, apricot blackberry , peach, peach, sapodilla, mamey, plum, jobo, guava, lime, lemon, tangerine, nectarine, tangerine, grapefruit, mango, apple, perón, melon, orange , papaya, pear, pineapple, pitahaya, prickly pear, plantain, green plantain, tabasco platain, other bananas (Chiapas, dominico, guineo, manzano, dorado, portalimón and roatán), watermelon, grape, garambullo, pomegranate, jicama, kiwi, fruit arrangement. |
| **Vegetables** | Chard, spinach, purslane, quintolines, quelites, pipica, avocado, garlic, broccoli, zucchini. squash, onion, chayote, pea, jalapeño chile, poblano chile, serrano chile, other chiles, cilantro, cabbage, corn, epazote, tomato, lettuce, nopal, cucumber, parsley, mint, green tomato, carrot, other vegetables, papalo, olives, pumpkin flower, chaya, corn sprouts, soybeans, wheat, alfalfa, packaged chili peppers, dried chili or in powder, packaged vegetables and legumes, olives, pickled vegetables, frozen vegetables and legumes, fresh mushrooms: mushrooms, huitlacoche and mushrooms. |
| **Legumes** | Beans in grain, chickpea in grain, yellow or green bean in grain, lentil in grain, other legumes in grain, processed beans, other processed legumes. |
| **Cereals and seeds** | Wheat flour, flour tortilla, pasta for soup, sweet cookies, salty crackers, white bread (bolillo, telera, baguette), sweet bread in pieces (of all kinds), bread for sandwiches, hamburgers, hot dogs and toasts, wheat grain, other wheat products (except cereal), rice grain, other rice products, corn grain (all types and colors), corn flour, cornstarch, starch, nixtamalized (for tortillas, for atole and flour for tamales), corn dough (all types and colors), corn tortillas (all types and colors), toasts, raspadas, tostitos, tortilla chips, tlayudas, other corn products (except cereal), seed, packed seeds, processed seeds and texturized soybeans. |
| **Egg** | Chicken egg |
| **Dairy** | Pasteurized cow's milk, evaporated milk, whole or skimmed milk powder, modified or formulated milk, unpasteurized milk (raw milk), other milks (donkey, goat, soy), yellow cheese in slices or spread, aged cheese, cotija cheese, chihuahua cheese, fresh cheese, manchego cheese, oaxaca or asadero cheese, other cheeses, cream, butter, fermented milk drinks, other milk derivatives. |
| **Meats** | Beef steak (from any part that is taken), flank steak, fillet, beef milanesa, beef chamorro, beef rib chop, needles, skirt, shank, backbone, beef fajilla for roasting, retazo, tampiqueña, beef stew, special cuts of beef, beef patties for grilling, ground beef, beef chunks, meat from other parts of beef, bone-in chicken leg, thigh or breast, chicken leg, thigh or breast boneless, whole chicken or pieces, viscera and other parts of chicken, other birds, lamb and mutton, goat and kid, other meats (horse, rabbit, iguana, wild boar, frog, turtle , deer) insects (grasshoppers, chinicuiles, escamoles, maguey worms, chicatana ants, jumiles). Cleaned and uncleaned whole fish, fish fillet, canned tuna, eels, eels, fish roe, stingray, alligator, fresh shrimp, fresh seafood, processed seafood. |
| **Fish and seafood** | Cleaned and uncleaned whole fish, fish fillet, canned tuna, eels, eels, fish roe, stingray, alligator, fresh shrimp, fresh seafood, processed seafood. |
| **Processed meat** | Enchilada meat, chicharron, chorizo with any seasoning and color and sausage, smoked pork chop, machaca and dried meat, pork ham, mortadella, pork cheese and salami, assorted meat bologna, bacon, sausages, salchichón, other processed meats, cueritos, chicken chorizo, ham and nugget , sausage, mortadella, salmon, processed cod, smoked, dried fish, nugget , sardine. |
| **Prepared foods** | Pizza, carnitas, roasted chicken, barbacoa, birria, atole, flautas, stews, hot dogs, soups, tacos, tamales, tortas, sopes, menudo, pozole, smoothies, jellies, corn, and other prepared foods. |
| **Taxed food and beverages** | Prepared water, natural juices, packaged juices, nectars, cola and flavored soft drinks, energy drinks, flans, jellies, powdered puddings, cajetas, milk sweets, hams, custards, fruit pastes, peanut butter, jellies, jam, ice creams, ice popsicles, other sweets, basket of sweets, sweet cookies, salty cookies, bread sweet in pieces (of all types), packaged sweet bread, cakes and pastries in pieces or in bulk, homemade cake, packaged cakes and pastries, corn, wheat, rice, oat, granola, snacks (chips, popcorn, cheetos, doritos), instant soups, flour for mashed potatoes, french fries in bags or in bulk, fruits in syrup and preserves, crystallized and dried fruits, condensed milk. |
| **Water** | Natural bottled water, mineral water, tonic water, demineralized with or without flavor |
| **Other food** | Butter, other types of eggs, vegetable oil, coconut oil and other oils, margarine, pork lard, vegetable hydrogenated oil, other oils, refined sugar, honey, other sugars, flowers for tea, soluble tea, powdered chocolate, other chocolates, cinnamon, cloves, fine herbs, chicken, tomato, shrimp concentrates, mayo, mustard, pepper, salt, spicy and sweet sauces, vinegar, others dressings. |

**Supplementary Table 2.** Demand system estimates.

|  | **alpha** | **beta** |
| --- | --- | --- |
| Fruits | -0.01 | 2.68 |
| Vegetables | -0.04* | -1.17 |
| Legumes | 0.05* | 8.51 |
| Cereals and seeds | 0.71* | -11.56 |
| Egg | 0.2* | 1.11 |
| Dairy | 0.18* | 1.34 |
| Unprocessed meats | -0.47* | 0.52* |
| Fish and seafood | -0.08* | -2.93 |
| Water | 0.14* | -4.47 |
| Processed meats | -0.09* | 11.61 |
| Prepared foods | -0.1* | -3.55 |
| Taxed food and beverages | 0.59* | 0.69 |
| Other foods | -0.09* | -2.77* |
| ∑ | 1.0 | 0.0 |

Own estimations using the 2018 MNHIES.

Estimates fulfill additivity $\sum_{i=1}^{n} \alpha_{i}=1$, $\sum_{i=1}^{n} \gamma_{i}=0$, $\sum_{i=1}^{n} \beta_{i}=0$, homogeneity $\sum_{i=1}^{n} \gamma_{ij}=0$ and symmetry $\gamma_{ij}=\gamma_{ji}$ restrictions imposed by the model.

| **gamma** | | | | | |
| --- | --- | --- | --- | --- | --- |
| Fruits_Fruits | 0.0110927 | Vegetables_Vegetables | -0.0115814 | Legumes_Legumes | 0.0158276 |
| Fruits_Vegetables | -0.0087619 | Vegetables_Legumes | 0.0131939 | Legumes_Cereals | -0.0121811 |
| Fruits_Legumes | 0.0029039 | Vegetables_Cereals | -0.0102438 | Legumes_Egg | -0.0055111 |
| Fruits_Cereals | 0.0011298 | Vegetables_Egg | 0.010719 | Legumes_Dairy | 0.0023424 |
| Fruits_Egg | -0.003789 | Vegetables_Dairy | -0.0083673 | Legumes_Unprocessed meats | 0.0190781 |
| Fruits_Dairy | 0.0025108 | Vegetables_Unprocessed meats | -0.0227349 | Legumes_Fish and seafood | 0.0009737 |
| Fruits_Unprocessed meats | -0.0256472 | Vegetables_Fish and seafood | 0.0123219 | Legumes_Water | 0.0028228 |
| Fruits_Fish and seafood | 0.0044407 | Vegetables_Water | 0.0052429 | Legumes_Processed meats | 0.0003204 |
| Fruits_Water | 0.0028735 | Vegetables_Processed meats | 0.0019902 | Legumes_Prepared foods | -0.0067372 |
| Fruits_Processed meats | -0.0001791 | Vegetables_Prepared foods | -0.0049672 | Legumes_Taxed food and beverages | -0.0223059 |
| Fruits_Prepared foods | -0.0009418 | Vegetables_Taxed food and beverages | 0.0442998 | Legumes_Other foods | -0.0107275 |
| Fruits_Taxed food and beverages | 0.0249211 | Vegetables_Other foods | -0.0211111 |  |  |
| Fruits_Other foods | -0.0105537 |  |  |  |  |
| **∑** | 0.0 |  | 0.0 |  | 0.0 |
| **gamma** | | | | | |
| Cereals_Cereals | 0.0346867 | Egg_Egg | 0.0096241 | Dairy_Dairy | -0.034155 |
| Cereals_Egg | 0.002556 | Egg_Dairy | 0.0099263 | Dairy_Unprocessed meats | 0.0120986 |
| Cereals_Dairy | 0.0055904 | Egg_Unprocessed meats | -0.0076974 | Dairy_Fish and seafood | 0.0020946 |
| Cereals_Unprocessed meats | 0.0153718 | Egg_Fish and seafood | -0.0004952 | Dairy_Water | 0.0004943 |
| Cereals_Fish and seafood | 0.0019791 | Egg_Water | 0.0000491 | Dairy_Processed meats | -0.0107222 |
| Cereals_Water | -0.0144832 | Egg_Processed meats | -0.006584 | Dairy_Prepared foods | 0.0015809 |
| Cereals_Processed meats | 0.0109471 | Egg_Prepared foods | 0.0015562 | Dairy_Taxed food and beverages | -0.0025497 |
| Cereals_Prepared foods | 0.0088099 | Egg_Taxed food and beverages | -0.0051302 | Dairy_Other foods | 0.019156 |
| Cereals_Taxed food and beverages | -0.014955 | Egg_Other foods | -0.0052237 |  |  |
| Cereals_Other foods | -0.0292076 |  |  |  |  |
| **∑** | 0.0 |  | 0.0 |  | 0.0 |
| **gamma** | | | | | |
| Unprocessed meats_Unprocessed meats | 0.00018 | Fish and seafood_Fish and seafood | -0.0108416 | Water_Water | -0.0105592 |
| Unprocessed meats_Fish and seafood | 0.0000228 | Fish and seafood_Water | -0.0025688 | Water_Processed meats | 0.0000465 |
| Unprocessed meats_Water | 0.0016345 | Fish and seafood_Processed meats | 0.001803 | Water_Prepared foods | 0.0052756 |
| Unprocessed meats_Processed meats | 0.0059928 | Fish and seafood_Prepared foods | -0.0067185 | Water_Taxed food and beverages | 0.0058199 |
| Unprocessed meats_Prepared foods | -0.0067369 | Fish and seafood_Taxed food and beverages | 0.0011671 | Water_Other foods | 0.0033523 |
| Unprocessed meats_Taxed food and beverages | 0.0169798 | Fish and seafood_Other foods | -0.0041788 |  |  |
| Unprocessed_Other foods | -0.0085419 |  |  |  |  |
| **∑** | 0.0 |  | 0.0 |  | 0.0 |

Own estimations using the 2018 MNHIES.

Estimates fulfill additivity $\sum_{i=1}^{n} \alpha_{i}=1$, $\sum_{i=1}^{n} \gamma_{i}=0$, $\sum_{i=1}^{n} \beta_{i}=0$, homogeneity $\sum_{i=1}^{n} \gamma_{ij}=0$ and symmetry $\gamma_{ij}=\gamma_{ji}$ restrictions imposed by the model.

**Supplementary Table 3.** Demand system estimates for covariates.

| **Eta** | | | | | | | | |
| --- | --- | --- | --- | --- | --- | --- | --- | --- |
|  | **Household size** | **Children 0 to 1 years** | **Children 2-5 years** | **Children 6-13 years old** | **Males 14-18 years old** | **Females 14-18 years old** | **Males >18 years old** | **Women >18 years old** |
| Fruits | -0.00033 | 0.00011 | 0.00002 | -0.00017 | -0.00037 | -0.00007 | -0.00101 | 0.00013 |
| Vegetables | 0.00028 | -0.00060 | -0.00089 | -0.00102 | -0.00024 | 0.00003 | -0.00009 | 0.00245 |
| Legumes | 0.00015 | -0.00019 | -0.00021 | -0.00009 | 0.00004 | -0.00008 | 0.00023 | 0.00027 |
| Cereals and seeds | 0.00084 | -0.00099 | -0.00139 | -0.00019 | -0.00021 | -0.00074 | 0.00084 | 0.00308 |
| Egg | 0.00017 | -0.00008 | -0.00008 | 0.00010 | 0.00013 | -0.00009 | 0.00011 | 0.00021 |
| Dairy | -0.00039 | 0.00226 | 0.00140 | 0.00095 | -0.00027 | 0.00003 | -0.00124 | 0.00071 |
| Unprocessed meats | 0.00045 | -0.00086 | -0.00075 | -0.00144 | 0.00028 | 0.00078 | 0.00104 | 0.00254 |
| Fish and seafood | -0.00010 | -0.00009 | 0.00002 | -0.00019 | 0.00009 | 0.00001 | -0.00001 | -0.00036 |
| Water | -0.00007 | -0.00008 | -0.00030 | -0.00010 | -0.00032 | -0.00026 | -0.00039 | 0.00020 |
| Processed meats | 0.00006 | -0.00019 | 0.00036 | 0.00021 | 0.00032 | 0.00023 | 0.00007 | -0.00061 |
| Prepared foods | -0.00065 | 0.00032 | 0.00112 | 0.00073 | 0.00062 | 0.00067 | 0.00034 | -0.00527 |
| Taxed food and beverages | -0.00047 | 0.00059 | 0.00080 | 0.00129 | -0.00013 | -0.00068 | -0.00009 | -0.00335 |
| Other foods | 0.00006 | -0.00018 | -0.00010 | -0.00008 | 0.00007 | 0.00018 | 0.00021 | 0.00000 |

Own estimations using the 2018 MNHIES.

| **Eta** | | | | | | | | | | | | |
| --- | --- | --- | --- | --- | --- | --- | --- | --- | --- | --- | --- | --- |
|  | **No schooling** | **Primary** | **Secondary** | **High school** | **University or higher** | **Very low** | **Low** | **Medium** | **High** | **Very high** | **Rural** | **Urban** |
| **Fruits** | -8.39 | -8.39 | -8.39 | -8.39 | -8.39 | 25.81 | 25.81 | 25.81 | 25.81 | 25.81 | -20.08 | -20.08 |
| **Vegetables** | 1.54 | 1.54 | 1.54 | 1.54 | 1.54 | -0.16 | -0.16 | -0.16 | -0.17 | -0.17 | -0.20 | -0.20 |
| **Legumes** | -0.29 | -0.29 | -0.29 | -0.29 | -0.29 | -6.92 | -6.92 | -6.92 | -6.92 | -6.92 | -1.30 | -1.30 |
| **Cereals and seeds** | 16.31 | 16.31 | 16.31 | 16.31 | 16.31 | -1.59 | -1.59 | -1.59 | -1.59 | -1.59 | -3.22 | -3.22 |
| **Egg** | 14.92 | 14.92 | 14.92 | 14.92 | 14.92 | -11.36 | -11.36 | -11.36 | -11.36 | -11.36 | -4.68 | -4.68 |
| **Dairy** | -7.68 | -7.68 | -7.68 | -7.68 | -7.68 | 9.98 | 9.98 | 9.98 | 9.98 | 9.98 | -3.65 | -3.65 |
| **Unprocessed meats** | 1.62 | 1.62 | 1.62 | 1.62 | 1.62 | -0.83 | -0.83 | -0.83 | -0.83 | -0.83 | -1.26 | -1.26 |
| **Fish and seafood** | 17.57 | 17.57 | 17.57 | 17.57 | 17.57 | -9.21 | -9.21 | -9.21 | -9.21 | -9.21 | -5.43 | -5.43 |
| **Water** | 5.95 | 5.95 | 5.95 | 5.95 | 5.95 | 7.11 | 7.11 | 7.11 | 7.11 | 7.11 | -8.60 | -8.60 |
| **Processed meats** | -10.59 | -10.59 | -10.59 | -10.59 | -10.59 | 6.16 | 6.16 | 6.16 | 6.16 | 6.16 | -7.17 | -7.17 |
| **Prepared foods** | -1.33 | -1.33 | -1.33 | -1.33 | -1.33 | 19.12 | 19.12 | 19.12 | 19.12 | 19.12 | -14.21 | -14.21 |
| **Taxed food and beverages** | 3.39 | 3.39 | 3.39 | 3.39 | 3.39 | -1.61 | -1.61 | -1.61 | -1.61 | -1.61 | -2.50 | -2.50 |
| **Other foods** | -33.02 | -33.02 | -33.02 | -33.02 | -33.02 | -36.49 | -36.49 | -36.49 | -36.49 | -36.49 | 72.30 | 72.30 |

Own estimations using the 2018 MNHIES

**Supplementary Table 4.** Own- and cross-price elasticities (95% CI).

|  | **Fruits** | **Vegetables** | **Legumes** | **Cereals and seeds** | **Egg** | **Dairy** | **Unprocessed meats** | **Fish and seafood** | **Water** | **Processed meats** | **Prepared foods** | **Taxed food and beverages** | **Other foods** |
| --- | --- | --- | --- | --- | --- | --- | --- | --- | --- | --- | --- | --- | --- |
| **Fruits** | -0.74 (-0.79 to -0.70) | -0.17 (-0.22 to -0.12) | 0.06 (0.03 to 0.08) | -0.1 (-0.14 to -0.06) | -0.11 (-0.15 to -0.08) | 0.03 (0.00 to 0.07) | -0.51 (-0.56 to -0.45) | 0.12 (0.09 to 0.14) | 0.04 (0.02 to 0.06) | 0.01 (-0.03 to 0.04) | -0.01 (-0.03 to 0.01) | 0.45 (0.40 to 0.50) | -0.23 (-0.27 to -0.19) |
| **Vegetables** | -0.06 (-0.08 to -0.04) | -1.09 (-1.13 to -1.05) | 0.11 (0.09 to 0.12) | -0.18 (-0.20 to -0.16) | 0.06 (0.04 to 0.08) | -0.09 (-0.11 to -0.07) | -0.14 (-0.17 to -0.11) | 0.12 (0.10 to 0.13) | 0.02 (0.01 to 0.03) | 0.03 (0.01 to 0.05) | -0.02 (-0.03 to -0.01) | 0.3 (0.28 to 0.33) | -0.17 (-0.19 to -0.15) |
| **Legumes** | 0.12 (0.08 to 0.17) | 0.53 (0.46 to 0.60) | -0.37 (-0.42 to -0.31) | -0.38 (-0.43 to -0.33) | -0.2 (-0.25 to -0.16) | 0.11 (0.06 to 0.15) | 0.72 (0.65 to 0.79) | 0.03 (0.00 to 0.06) | 0.13 (0.11 to 0.16) | 0 (-0.05 to 0.06) | -0.29 (-0.31 to -0.27) | -0.87 (-0.93 to -0.81) | -0.43 (-0.49 to -0.37) |
| **Cereals and seeds** | -0.01 (-0.02 to 0.00) | -0.11 (-0.13 to -0.09) | -0.07 (-0.08 to -0.06) | -0.39 (-0.42 to -0.36) | 0.11 (0.09 to 0.12) | 0.12 (0.10 to 0.14) | -0.08 (-0.11 to -0.05) | -0.02 (-0.03 to -0.01) | -0.05 (-0.06 to -0.04) | 0.06 (0.05 to 0.08) | 0.02 (0.01 to 0.03) | 0.13 (0.11 to 0.15) | -0.3 (-0.32 to -0.29) |
| **Egg** | -0.11 (-0.15 to -0.08) | 0.24 (0.19 to 0.30) | -0.13 (-0.16 to -0.10) | 0.32 (0.28 to 0.36) | -0.69 (-0.75 to -0.64) | 0.31 (0.27 to 0.35) | -0.36 (-0.42 to -0.31) | -0.04 (-0.06 to -0.01) | 0.06 (0.04 to 0.08) | -0.19 (-0.23 to -0.15) | 0 (-0.02 to 0.02) | 0.07 (0.02 to 0.11) | -0.18 (-0.23 to -0.13) |
| **Dairy** | 0.03 (0.01 to 0.04) | -0.09 (-0.12 to -0.07) | 0.03 (0.01 to 0.04) | 0.11 (0.09 to 0.14) | 0.12 (0.10 to 0.13) | -1.34 (-1.37 to -1.31) | 0.09 (0.06 to 0.12) | 0.01 (0.00 to 0.02) | 0.02 (0.01 to 0.03) | -0.12 (-0.13 to -0.10) | 0.01 (-0.01 to 0.02) | 0.02 (-0.01 to 0.05) | 0.19 (0.17 to 0.21) |
| **Unprocessed meats** | -0.13 (-0.14 to -0.11) | -0.1 (-0.12 to -0.09) | 0.09 (0.08 to 0.10) | -0.13 (-0.15 to -0.12) | -0.1 (-0.11 to -0.08) | 0.01 (0.00 to 0.03) | -0.87 (-0.90 to -0.84) | 0.03 (0.02 to 0.04) | -0.04 (-0.05 to -0.03) | 0.05 (0.04 to 0.07) | 0 (-0.01 to 0.01) | -0.07 (-0.09 to -0.05) | 0 (-0.02 to 0.01) |
| **Fish and seafood** | 0.23 (0.18 to 0.28) | 0.6 (0.53 to 0.67) | 0.02 (-0.02 to 0.05) | -0.22 (-0.28 to -0.16) | -0.1 (-0.14 to -0.05) | 0.01 (-0.05 to 0.06) | 0.19 (0.11 to 0.28) | -1.45 (-1.50 to -1.40) | -0.19 (-0.22 to -0.16) | 0.1 (0.05 to 0.15) | -0.27 (-0.30 to -0.24) | -0.19 (-0.26 to -0.12) | -0.14 (-0.20 to -0.09) |
| **Water** | 0.26 (0.16 to 0.35) | 0.48 (0.35 to 0.62) | 0.41 (0.34 to 0.48) | -0.52 (-0.65 to -0.40) | 0.29 (0.21 to 0.38) | 0.34 (0.23 to 0.45) | -0.52 (-0.69 to -0.35) | -0.43 (-0.50 to -0.35) | -1.92 (-2.01 to -1.84) | -0.09 (-0.19 to 0.01) | 0.42 (0.35 to 0.49) | 1.54 (1.39 to 1.69) | 0.14 (0.04 to 0.24) |
| **Processed meats** | 0.01 (-0.02 to 0.04) | 0.06 (0.01 to 0.10) | 0 (-0.03 to 0.02) | 0.07 (0.04 to 0.11) | -0.16 (-0.19 to -0.13) | -0.23 (-0.27 to -0.20) | 0.21 (0.16 to 0.25) | 0.05 (0.03 to 0.07) | -0.03 (-0.05 to -0.01) | -0.72 (-0.77 to -0.68) | 0.02 (0.00 to 0.03) | -0.54 (-0.58 to -0.50) | 0.11 (0.08 to 0.15) |
| **Prepared foods** | 0 (-0.01 to 0.01) | -0.04 (-0.05 to -0.02) | -0.08 (-0.09 to -0.08) | -0.04 (-0.06 to -0.03) | -0.02 (-0.02 to -0.01) | -0.02 (-0.03 to 0.00) | 0.01 (0.00 to 0.03) | -0.06 (-0.06 to -0.05) | 0.02 (0.02 to 0.03) | 0.01 (0.00 to 0.02) | -1.03 (-1.05 to -1.01) | -0.09 (-0.11 to -0.08) | 0.16 (0.15 to 0.17) |
| **Taxed food and beverages** | 0.19 (0.18 to 0.21) | 0.35 (0.33 to 0.38) | -0.18 (-0.19 to -0.17) | 0.12 (0.09 to 0.14) | 0.02 (0.01 to 0.04) | 0.04 (0.02 to 0.06) | 0 (-0.04 to 0.03) | -0.02 (-0.03 to -0.01) | 0.11 (0.09 to 0.12) | -0.22 (-0.24 to -0.20) | -0.03 (-0.04 to -0.02) | -1.29 (-1.33 to -1.25) | 0.2 (0.18 to 0.22) |
| **Other foods** | -0.18 (-0.21 to -0.15) | -0.36 (-0.41 to -0.31) | -0.2 (-0.22 to -0.17) | -0.71 (-0.75 to -0.68) | -0.14 (-0.17 to -0.11) | 0.29 (0.26 to 0.32) | 0.01 (-0.04 to 0.05) | -0.05 (-0.07 to -0.03) | 0.01 (-0.01 to 0.02) | 0.1 (0.07 to 0.14) | 0.26 (0.25 to 0.28) | 0.36 (0.32 to 0.40) | -0.57 (-0.63 to -0.52) |

Own estimations using the 2018 MNHIES.

Gray values correspond to own-price elasticities.

**Supplementary Table 5.** Mean own-price elasticities of food group, otherwise ranges in parenthesis.

| **Food group** | **Own-price elasticity** | **Country or countries** |
| --- | --- | --- |
| Fruits^(1)^ | (−0.41,−0.98) | USA |
| Fruits^(2)^ | −0.52 | USA |
| Fruits^(3)^ | −0.65 | USA |
| Fruits^(4)^ | −0.713 | USA |
| Fruits^(5)^ | −0.41 | Finland |
| Fruits^(6)^ | −0.595 | India |
| Fruits^(7)^ | −0.34 | USA |
| Fruits^(8)^ | (−0.32,−0.34) | Latin America/ Caribbean |
| Fruits and vegetables^(9)^ | (−1.04, −0.13) | Mexico, South Africa, USA, Hungary, Australia, Denmark, India, Finland, UK, Latvia |
| Fruits and vegetables^(10)^ | −0.72 | Côte d'Ivoire, Egypt, Ghana, Tanzania, Uganda, Bangladesh, Pakistan, Philippines, Vietnam, Bolivia, Ecuador, Paraguay |
| Fruits and vegetables ^(10)^ | −0.64 | South Africa, Turkey, Saudi Arabia, Bulgaria, Hungary, Latvia, Lithuania, Romania, Slovenia, Mexico, Brazil |
| Fruits and vegetables ^(10)^ | −0.53 | Australia, Japan, Taiwan, Denmark, Finland, France, Germany, Italy, Norway, Spain, Switzerland, UK, Canada, USA |
| Fruits and vegetables^(11)^ | −0.35  (−0.21,−0.77) | France, USA, Ireland, UK, Norway, Denmark, Sweden, Australia |
| Fruits and vegetables ^(10)^ | −0.73  (−0.84, −0.62) | 167 European and Asian countries predominantly |
| Vegetables^(1)^ | −0.58  (−0.44, −0.71) | USA |
| Vegetables^(2)^ | −0.69 | USA |
| Vegetables^(3)^ | −0.7 | USA |
| Vegetables^(4)^ | −0.713 | USA |
| Vegetables^(5)^ | −0.41 | Finland |
| Vegetables^(6)^ | −0.515 | India |
| Vegetables^(8)^ | −0.32 | USA |
| Legumes^(6)^ | −0.635 | India |
| Legumes | −0.453 | India |
| Legumes^(8)^ | (−0.17,−0.07) | Latin America/ Caribbean |
| Cereals^(1)^ | −0.60  (−0.43, −0.77) | USA |
| Cereals ^(10)^ | −0.61 | Côte d'Ivoire, Egypt, Ghana, Tanzania, Uganda, Bangladesh, Pakistan, Philippines, Vietnam, Bolivia, Ecuador, Paraguay |
| Cereals ^(10)^ | −0.55 | South Africa, Turkey, Saudi Arabia, Bulgaria, Hungary, Latvia, Lithuania, Romania, Slovenia, Mexico, Brazil |
| Cereals ^(10)^ | −0.43 | Australia, Japan, Taiwan, Denmark, Finland, France, Germany, Italy, Norway, Spain, Switzerland, UK, Canada, USA |
| Cereals ^(10)^ | −0.72  (−0.85, −0.59) | 167 European and Asian countries predominantly |
| Cereals^(6)^ | −0.031 | India |
| Egg^(1)^ | −0.27  (−0.08, −0.45) | USA |
| Egg^(4)^ | −0.86 | USA |
| Egg^(12)^ | −1.72 | Bangladesh |
| Conventional egg^(13)^ | −0.2348 | USA |
| Dairy^(1)^ | −0.65  (−0.46, −084) | USA |
| Dairy^(14)^ | −0.78 | Côte d'Ivoire, Egypt, Ghana, Tanzania, Uganda, Bangladesh, Pakistan, Philippines, Vietnam, Bolivia, Ecuador, Paraguay |
| Dairy ^(14)^ | −0.72 | South Africa, Turkey, Saudi Arabia, Bulgaria, Hungary, Latvia, Lithuania, Romania, Slovenia, Mexico, Brazil |
| Dairy ^(14)^ | −0.6 | Australia, Japan, Taiwan, Denmark, Finland, France, Germany, Italy, Norway, Spain, Switzerland, UK, Canada, USA |
| Milk^(1)^ | −0.59  (−0.40, −0.79) | USA |
| Milk | −1.035 | India |
| Milk^(6)^ | −0.624 | India |
| Milk^(12)^ | −2.14 | Bangladesh |
| Milk^(8)^ | −0.24 | Latin America/ Caribbean |
| Carne ^(14)^ | −0.78 | Côte d'Ivoire, Egypt, Ghana, Tanzania, Uganda, Bangladesh, Pakistan, Philippines, Vietnam, Bolivia, Ecuador, Paraguay |
| Meat ^(14)^ | −0.72 | South Africa, Turkey, Saudi Arabia, Bulgaria, Hungary, Latvia, Lithuania, Romania, Slovenia, Mexico, Brazil |
| Meat ^(14)^ | −0.6 | Australia, Japan, Taiwan, Denmark, Finland, France, Germany, Italy, Norway, Spain, Switzerland, UK, Canada, USA |
| Meat ^(14)^ | −0.95  (−1.07, −0.82) | 167 European and Asian countries predominantly |
| Non-processed meat^(8)^ | (−0.21, −0.18) | Latin America/ Caribbean |
| Processed meat^(8)^ | (−0.3, −0.22) | Latin America/ Caribbean |
| Processed meat^(15)^ | −0.03 | Canada |
| Water^(16)^ | (−0.2, −0.3) | USA |
| Water^(17)^ | (−0.16, −0.39) | USA |
| Water^(18)^ | −0.15 | USA |
| Water^(19)^ | −0.41 | USA |
| Water^(20)^ | (−0.13, −1.93) | USA |
| Water^(21)^ | −0.36 | USA |
| Water^(22)^ | −0.12 | USA |
| Water^(23)^ | −0.34 | USA |
| Water^(24)^ | (−0.43, −1.14) | USA |
| Water^(25)^ | (−0.15, −0.30) | USA |
| Water^(26)^ | -0.66 | USA |

**References**

1. Andreyeva T, Long MW & Brownell KD (2010) The impact of food prices on consumption: A systematic review of research on the price elasticity of demand for food. *Am J Public Health* **100**, 216–222.

2. Dong D & Lin BH (2011) Consumption by low-income Americans: Would a price reduction make a difference? *Eating Right: The Consumption of Fruits and Vegetables*, 245–261.

3. Huang KS & Lin B-H *Demand and Nutrient Household Survey Data*. .

4. Jensen JD & Smed S (2007) Cost-effective design of economic instruments in nutrition policy. *International Journal of Behavioral Nutrition and Physical Activity* **4**, 1–12.

5. Kotakorpi K, Härkänen T, Pietinen P, et al. (2011) The Welfare Effects of Health-based Food Tax Policy. .

6. Kumar P, Kumar A, Parappurathu S, et al. (2011) Estimation of Demand Elasticity for Food Commodities in India. *Agricultural Economics Research Review* **24**, 1–14.

7. Park JL, Holcomb RB, Raper KC, et al. (1996) *A Demand Systems Analysis of Food Commodities by U.S. Households Segmented by Income*. .

8. USDA (2017) *Own-price elasticity estimates of key food categories by region, sex, and age*. .

9. Andreyeva T, Marple K, Moore TE, et al. (2022) Evaluation of Economic and Health Outcomes Associated with Food Taxes and Subsidies: A Systematic Review and Meta-analysis. *JAMA Netw Open* **5**, E2214371.

10. Green R, Cornelsen L, Dangour AD, et al. (2013) The effect of rising food prices on food consumption:systematic review with meta-regression. *BMJ (Online)* **347**, 1–9.

11. Eyles H, Ni Mhurchu C, Nghiem N, et al. (2012) Food Pricing Strategies, Population Diets, and Non-Communicable Disease: A Systematic Review of Simulation Studies. *PLoS Med* **9**.

12. Al Sayed MAU, Das S, Akter F, et al. (2019) An investigation on price elasticity of demand for protein consumption in Sylhet City, Bangladesh. *Munich Personal RePEc Archive*.

13. Bakhtavoryan R, Hovhannisyan V, Devadoss S, et al. (2021) An Empirical Evaluation of Egg Demand in the United States. *Journal of Agricultural and Applied Economics* **53**, 280–300.

14. Cornelsen L, Green R, Turner R, et al. (2015) What happens to patterns of food consumption when food prices change? Evidence from a systematic review and meta-analysis of food price elasticities globally. *Health Economics (United Kingdom)* **24**, 1548–1559.

15. Irié II (2018) The Demand for Processed Meat in Canada: An Application of the Almost Ideal Demand System. .

16. Baerenklau KA, Schwabe KA & Dinar A (2014) The residential water demand effect of increasing block rate water budgets. *Land Econ* **90**, 683–699.

17. Browne OR, Gazze L & Greenstone M (2021) Do Conservation Policies Work? Evidence from Residential Water Use. *Environ Energy Policy Econ* **2**, 190–225.

18. Buck S, Auhammer M, Hamilton S, et al. (2015) *Measuring the Welfare Losses from Urban Water Supply Disruptions*. .

19. Dalhuisen JM, Florax RJGM, de Groot HLF, et al. (2003) Price and income elasticities of residential water demand: A meta-analysis. *Land Econ* **79**, 292–308.

20. Klaiber HA, Smith VK, Kaminsky M, et al. (2014) Measuring price elasticities for residential water demand with limited information. *Land Econ* **90**, 100–113.

21. Mansur ET & Olmstead SM (2012) The value of scarce water: Measuring the inefficiency of municipal regulations. *J Urban Econ* **71**, 332–346.

22. Nataraj S & Hanemann WM (2011) Does marginal price matter? A regression discontinuity approach to estimating water demand. *J Environ Econ Manage* **61**, 198–212.

23. Olmstead S, Hanemann WM & Stavins RN (2007) *Water Demand Under Alternative Price Structures*. .

24. Wichman CJ (2014) Perceived price in residential water demand: Evidence from a natural experiment. *J Econ Behav Organ* **107**, 308–323.

25. Wichman CJ, Taylor LO & von Haefen RH (2016) Conservation policies: Who responds to price and who responds to prescription? *J Environ Econ Manage* **79**, 114–134.

26. Yoo J, Simonit S, Kinzig AP, et al. (2014) Estimating the price elasticity of residential water demand: The case of Phoenix, Arizona. *Appl Econ Perspect Policy* **36**, 333–350.
